# The function and evolution of child-directed communication

**DOI:** 10.1371/journal.pbio.3001630

**Published:** 2022-05-06

**Authors:** Johanna Schick, Caroline Fryns, Franziska Wegdell, Marion Laporte, Klaus Zuberbühler, Carel P. van Schaik, Simon W. Townsend, Sabine Stoll

**Affiliations:** 1 Department of Comparative Language Science, University of Zurich, Zurich, Switzerland; 2 Center for the Interdisciplinary Study of Language Evolution (ISLE), University of Zurich, Zurich, Switzerland; 3 Department of Comparative Cognition, University of Neuchatel, Neuchatel, Switzerland; 4 Histoire naturelle de l’Homme préhistorique, UMR 7194, PaleoFED, Muséum National d’Histoire Naturelle, Paris, France; 5 Institut des Sciences du Calcul et des Données, Sorbonne Université, Paris, France; 6 School of Psychology and Neuroscience, University of St. Andrews, St. Andrews, United Kingdom; 7 Department of Anthropology, University of Zurich, Zurich, Switzerland; 8 Department of Evolutionary Biology and Environmental Studies, University of Zurich, Zurich, Switzerland; 9 Department of Psychology, University of Warwick, Warwick, United Kingdom

## Abstract

Humans communicate with small children in unusual and highly conspicuous ways (child-directed communication (CDC)), which enhance social bonding and facilitate language acquisition. CDC-like inputs are also reported for some vocally learning animals, suggesting similar functions in facilitating communicative competence. However, adult great apes, our closest living relatives, rarely signal to their infants, implicating communication surrounding the infant as the main input for infant great apes and early humans. Given cross-cultural variation in the amount and structure of CDC, we suggest that child-surrounding communication (CSC) provides essential compensatory input when CDC is less prevalent—a paramount topic for future studies.

## Abstract 2

Child-directed communication is considered essential for language acquisition, yet appears to be near-absent in some human cultures and in our closest-living relatives, the great apes. This Essay sheds light on the evolution of child-directed and child-surrounding communication.

## Introduction

Human languages exhibit enormous variation at all linguistic levels, ranging from phonemes, the smallest meaning-distinguishing units, to morphemes, the smallest meaning-bearing units, to words, higher-level constructions, and rules of combination. Few, if any, of these features are under strong genetic control. As a consequence, linguistic units must be learned from scratch by every maturing individual: a process that, while often described as “effortless” [1], in fact takes many thousands of hours of exposure over multiple years. Inevitably, the communicative environment must provide the input required for learning a native language.

One prominent source of this input is a special speech register used by caregivers to address infants and young children, frequently referred to as baby talk, motherese, parentese, and, more recently, infant-directed or child-directed speech [2]. In this Essay, we use a more neutral term child-directed communication (CDC; see Box 1) since there is lack of agreement of what constitutes infancy in humans, and moreover, the input is modality independent (i.e., it is also encountered in sign languages [3,4]). Such cross-modal prevalence has even been argued to support the notion that CDC is an automatic and potentially species-wide trait [5]. Both in signed and spoken languages, CDC includes other multimodal features such as more exaggerated facial expressions [6], modified gestures [7], and motions in general, with the latter known as motionese [8].

**Box 1. Definitions of key terms**

Child-directed communication (CDC): All communication specifically directed at children, in which the properties and structure of the signal often change in predictable ways, e.g., higher pitch, more exaggerated gestures, and more repetition. CDC supports language learning in children [2,9].

Child-surrounding communication (CSC): All communication that is perceptible to the child but not directed at them.

Immature-directed communication (IDC): All communication specifically directed at the immature animal, as indicated by the vocalizations or gestures being accompanied by body or head orientation toward the immature animal, as well as a change in structural or acoustic features, e.g., more repetition.

Natural pedagogy: The specific aspects of human communication that allow and facilitate the transfer of generic knowledge to novices [10].

Nine-month revolution: A large set of cognitive and sociocognitive skills that human infants typically develop at around 9 to 12 months of age. Within this skill set, they develop the ability to use gaze following, social referencing, pointing, joint attention, and imitation to join the adult’s attentional focus [11]. They also become able to interpret adults’ gestures as intentional acts [12].

Vocal learning: Describes vocal production learning, which is traditionally defined as the production of novel vocalizations as a result of learning from an acoustic signal [13]. Today, many dimensions and degrees of vocal production learning are acknowledged [14]. Only few animal species are known to be capable of vocal production learning (e.g., songbirds, hummingbirds, cetaceans, and pinnipeds). Besides vocal production learning there are usage and comprehension learning, which are known for most species [15]. Usage learning is defined as learning to produce a signal in a new context as a result of acoustic experience. Comprehension learning is defined as learning a new meaning of a signal as a result of experience [13].

A second and much less researched source of input is child-surrounding communication (CSC; Box 1), which includes all communication that is in perceptible proximity to, but not specifically directed toward the child. Typically, this involves 2 or more individuals engaged in some type of social interaction accompanied by a linguistic exchange. It may also include linguistic input from media sources (e.g., TV and radio), but it remains unclear which impact this type of input might have on the child’s language development. CSC input is ubiquitous, and at least as omnipresent as CDC, yet we know much less about its functional role in language acquisition. The few available studies on CSC suggest that it has less impact than CDC on linguistic development in early ontogeny [16,17].

**Fig 1:**
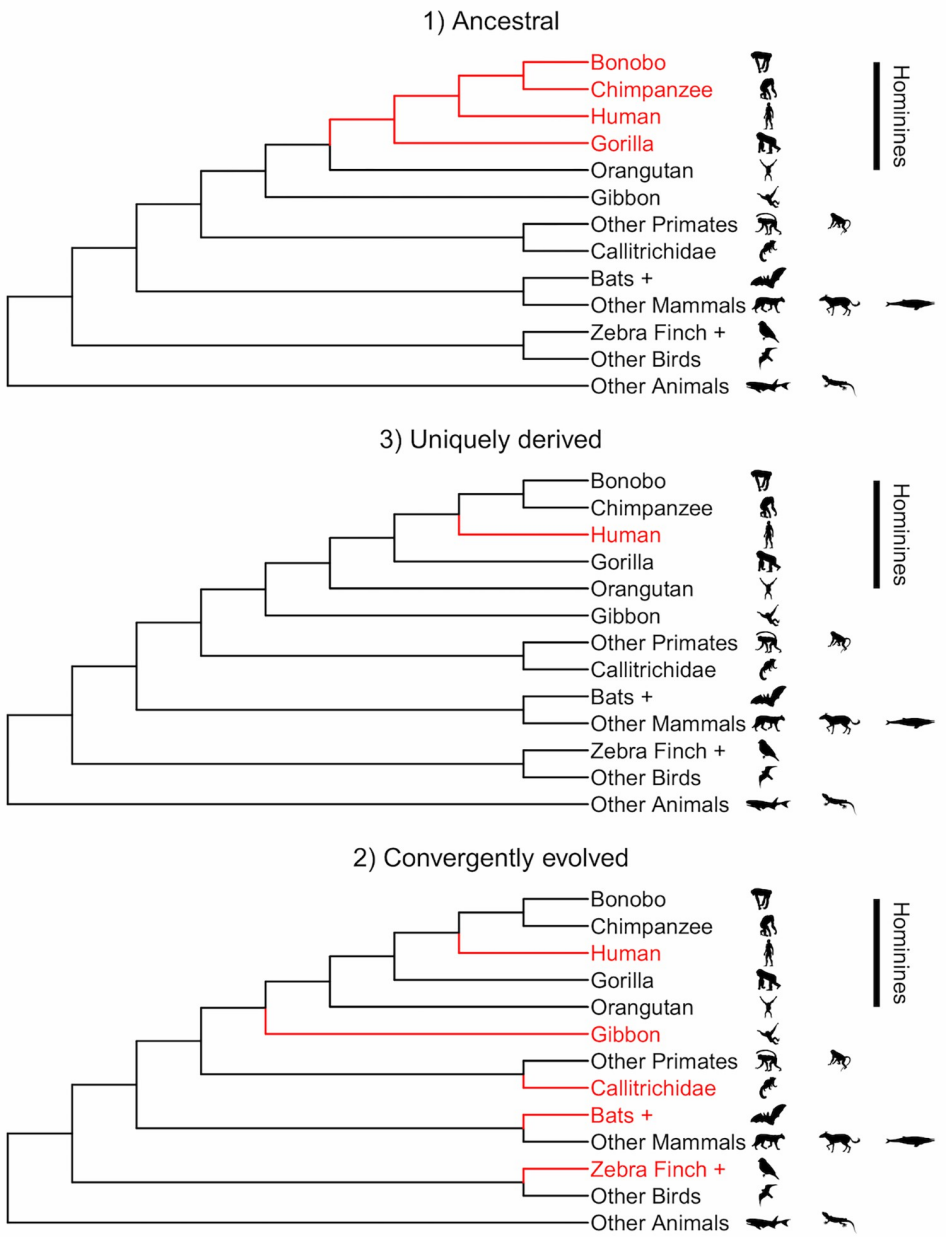
Evolutionary pathways of CDC. A feature such as CDC with the function of aiding the acquisition of communicative competence can be (1) ancestral: homologously derived among African great apes and thus also found in humans; (2) unique among the great apes but convergently shared analogously with other, more distantly related species; or (3) newly evolved within our own species. Red represents the presence of IDC features. *Outline credits*: *Human*: *T*. *Michael Keesey; Chimpanzee*: *Jonathan Lawley; Bonobo*: *T*. *Michael Keesey; Gorilla*: *T*. *Michael Keesey (after Colin M*.*L*. *Burnett); Orangutan*: *Gareth Monger; Gibbon*: *Kai R*. *Caspar; Tamarin*: *Yan Wong and T*.*F*. *Zimmerman; Zebra Finch*: *Jim Bendon (photography) and T*. *Michael Keesey (vectorization); Bat*: *Yan Wong; Squamate*: *Ghedo and T*. *Michael Keesey; Feline*: *Margot Michaud; Equine*: *T*. *Michael Keesey; Cetacean*: *Scott Hartman; Falcon*: *Liftarn; Fish*, *macaque and baboon are uncredited*. Link to creative commons license: https://creativecommons.org/licenses/by-sa/3.0. Link to public domain license: https://creativecommons.org/publicdomain/zero/1.0. Outlines were downloaded from http://www.http://phylopic.org. The layout of the figure was achieved in R (version 4.1.2, R Development Core Team, 2012). CDC, child-directed communication; IDC, immature-directed communication.

The reliance on CDC for the acquisition of communicative competence may be explained by 3 distinct evolutionary pathways (Fig 1). First, it might be shared with our closest living relatives, the great apes. If this is the case, we can assume that it is a feature that was also present in early hominins (i.e., the “African Apes”; extant and extinct *Homo*, *Pan*, and *Gorillini* genera). Second, it may be derived in humans and perhaps be one of the drivers of the evolution of language, potentially as part of a wider change in cognitive architecture of early humans. This derived state can have arisen uniquely in our ancestors or, third, it can be fully or partially shared with other, distantly related taxa, in which case it arose via convergent evolution.

Current evidence suggests that in nonhuman primates in general (hereafter primates), the ability to produce species-specific vocalizations develops with relatively little environmental contribution, i.e., irrespective of auditory input [18–21]. Instead, input seems to have more of a role in guiding vocal usage and comprehension [22–25]. Nonetheless, at least some vocal production, flexibility does exist in primates, although mainly in terms of socially driven vocal accommodation [22,26–32]. Although this suggests a role for social input, how much of this is immature-directed communication (IDC) versus immature-surrounding communication remains unclear [33]. So far, the few studies that have assessed immature-directed vocalizations in great apes have yielded low rates (chimpanzees, *Pan troglodytes* [33]; bonobos, *Pan paniscus* [34]). A few studies have described vocalizations used by mothers in chimpanzees [35] and orangutans [36]. However, this directed communication does not display any of the features or functions of natural pedagogy. Overall, the current state of the art suggests that immature-directed input has only a small impact on great ape vocal ontogeny, if any. The preliminary conclusion thus appears to be that most acoustic features of CDC are derived in humans. However, in the structural domain, some precursors of CDC might exist in apes.

However, a striking exception is found in the gestural domain. Orangutans [37], chimpanzees [38], and bonobos [39] all use immature-directed gestures. Furthermore, one CDC-like feature, repetition is found in gorilla [40] and chimpanzee gestures [41]. The use of specific gestures and their repetition rates by adult great apes toward immature individuals varies depending on the age and experience of the immature animal, as in humans, suggesting functional significance in the acquisition of communicative competence [40,41]. However, repetitions of gestures following lack of comprehension have also been described in adult orangutans [42]. In addition, bonobos modify communication signals according to recipient familiarity [43]. All of this suggests at least some shared cognitive features with humans. Evidently, more research is needed to assess whether immature-directed gestures can be considered the functional equivalent of CDC, especially in light of suggestions that at least part of the gestural repertoire are the result of innovations and therefore have to be learned [44].

**Fig 2:**
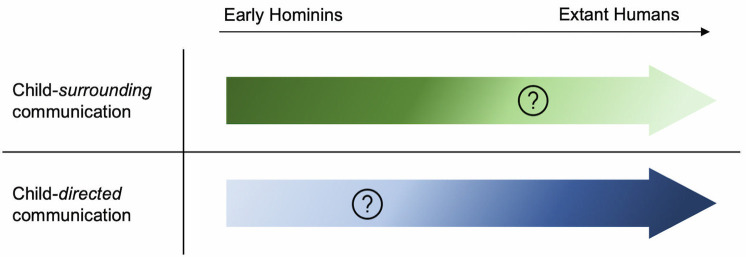
Transition of CSC to CDC. The transition of the importance of use of CSC to CDC. Darker color shows importance/presence and brighter color possible insignificance of CSC and CDC from early hominins to extant humans. CDC, child-directed communication; CSC, child-surrounding communication.

If CDC is fully or at least partially derived in humans, this raises 2 important questions. First, which elements of the broad bundle of features that make up human CDC were already present in the last common ancestor? Identifying which elements were preexisting (homologies: present in great apes), which are found in other animals (analogies: convergently evolved), and which are new and uniquely derived in our lineage would improve our understanding of how language acquisition evolved (Fig 1). Second, as IDC in primates in general appears to be rare, primates must acquire the learnt part of their communication from the communication that surrounds them, but is largely not directed at them. Has this originally predominant source of input remained significant in humans, or has CDC replaced it (Fig 2)?

In this Essay, we aim to address these 2 questions. In the first section, we deconstruct CDC into its component parts and assess their proposed functions; we then ask for each of them whether comparable phenomena exist in nonhuman animals (hereafter animals). In the second section, we contrast CDC in humans with the lesser-studied CSC to shed light on the interplay between these 2 forms of input and their respective roles in language acquisition. Answers to these questions should not only improve our understanding of the development and acquisition of language but also its evolutionary progression.

## The features and functions of CDC

CDC differs from adult-directed communication in a wide range of acoustic and structural features. This has been observed in numerous cultures and is widely considered a universal of human language [9,45,46]. Over the past few decades, a plethora of studies have shown that features of CDC (Table 1) support language acquisition by infants both in comprehension [47,48] and production [49–51]. CDC is part of a more general package of child-directed behaviors that serve to pass on cultural knowledge and skills to the next generation, known as natural pedagogy [10] (Box 1). This active transmission process rests on a (arguably) uniquely human capacity, ostension, which underlies pointing and results in gaze following (often followed by joint attention on objects between caretaker and child [52] or a state of shared intentionality more broadly [11]), as well as child-directed speech [10]. In this Essay, we argue that CDC is a crucial part of this universal form of teaching. Such natural pedagogy is almost certainly derived relative to the nonhuman great apes (hereafter great apes) and potentially evolved in relation to the frequently highlighted shift in the breeding system from independent to more cooperative [53]. Although the child-development literature may seem to suggest that natural pedagogy is primarily aimed at preverbal infants and mainly geared toward teaching cultural knowledge, CDC is an obvious and essential part of natural pedagogy extending well beyond early infancy. In fact, one might hypothesize that CDC is a core feature enabling the transmission of language and, as a consequence, the evolution of such a complex communication system.

**Table 1:** Known features of CDC.

Type of feature	Known feature of CDC	Proposed function	Reference
Acoustic	Pitch variability	Attention grabbing	[54]
Acoustic	Lengthening of vowels and pauses	Segmentation and discrimination of sounds	[55,56]
Acoustic	Extended vowel triangle	Sound discrimination	[57]
Acoustic	Clear articulation	Facilitate comprehension	[46,58]
Acoustic	Increased voice onset time	Sound discrimination	[59]
Acoustic	Slower speaking rate	Facilitate comprehension, discrimination, and segmentation	[54,60]
Structural	Frequent repetitions	Structural generalization of word/unit classes	[61,62]
Structural	Short utterances	Facilitate comprehension	[63,64]
Structural	Low type/token ratio	Facilitate comprehension	[65,66]
Structural	Simplified syntax and semantics	Facilitate comprehension	[63,65]
Structural	Frequent use of diminutives	Simplification of certain morphological aspects (language specific)	[67,68]
Structural	Frequent questions	Invite response, repetition, and attention grabbing	[69,70]
Structural	Variation sets	Structural generalization of word classes	[71,72]
Structural	Scaffolding	Learning of word constructions	[73]

The first 8 entries above the bold dividing line represent elements where a corresponding form could possibly be present in animal vocal communication.

CDC, child-directed communication.

Adults and older children use the bundle of acoustic and structural features of CDC in varying combinations when talking to infants and younger children (Table 1). For many of these features, there is evidence that they facilitate the child’s language learning.

Regarding the prosodic and acoustic features of the speech, CDC involves the production of higher and more variable pitch [54], systematic lengthening of vowels and pauses [55,56,74], and an extended “vowel triangle” or vowel hyperarticulation [57,75]. Studies have shown that these prosodic modifications attract the child’s attention [76] from an early age and that CDC is more salient to children than adult-directed communication and is actually preferred by them [60,77–79]. Indeed, neurobiological research has revealed that an infant’s exposure to CDC in their first year of life results in a higher brain activation in their left and right temporal areas compared with adult-directed speech [80]. These prosodic modifications also elicit increased infant vocal responses during their prelinguistic phase [81], a form of active participation crucial to language acquisition [2]. Infants listening to CDC rather than adult-directed speech also show greater sensitivity to syllable and vowel discrimination [75,82]. Last, caregivers tend to use exaggerated prosody to mark new or relevant vocabulary [74,83,84]. These prosodic characteristics of CDC not only support the detection of word boundaries [85], but also word comprehension [48,86] and production [49]. In sum, acoustic alternations of the speech signal appear to accelerate various aspects of language acquisition (see [87] for a review), suggesting that CDC serves as an evolved teaching tool.

Regarding the structural features, CDC is characterized by short utterances [63,64], a low type/token ratio [65,66], which indicates that caregivers use a simplified vocabulary, and the use of many questions [69,70], diminutives [67,68] and repetitions [61,62]. One structural feature in particular is known to have a significant role in the acquisition of language: frequency effects. The more frequently an element occurs in the child’s input, the faster it is expected to be learned [88,89]. Recent research has also shown that frequent repetitions are structured in CDC. Repetitions of constructions at the beginning of utterances (e.g., this is an X [62,90]) and discontinuous repetitions (e.g., I X you [91,92]) are ubiquitous and support the generalization of word classes, such as nouns and verbs [93]. In addition, repetitive structures or distribution of words surrounding specific verbs support the generalization of meaning [94], and the high number of repetitions found in CDC are positively correlated with word comprehension [95,96]. A specific form of repetitions frequently used in CDC is variation sets, successive utterances with partial self-repetitions produced by caregivers [71,72], which themselves are positively related to better linguistic outcomes in naturalistic longitudinal [97], and experimental settings [98]. These findings again support the hypothesis that CDC functions to accelerate language acquisition.

In addition to the prosodic and structural features of CDC, another important factor is the absolute amount of linguistic input children receive. A number of studies have indicated that the amount of CDC children experience is correlated with their later vocabulary development [16,99–102] and their word processing skills [101]. The quality (variety of words and syntactic structures) of CDC also impacts language development. Longitudinal studies have shown how input quality at an earlier stage of development predicts subsequent diversity and variance in language outcome at a later stage of development [103,104]. Quality and quantity may even have different roles during the child’s language development. For example, a longitudinal study of vocabulary acquisition revealed that input quantity mattered most during the second year of development, whereas input quality was more important during the third year [50]. The child’s ability to profit from different properties of CDC might therefore vary across development.

Most of the previously reviewed evidence is from children growing up in modern Western societies, characterized by child-rearing practices that are very different from what is typically seen in hunter-gatherer groups, our evolved and species-typical way of life [17]. In addition, there is substantial variation both within and across cultures in the amount of CDC that occurs and its features. Also important is that, in terms of sheer amount, there are linguistic communities in which children are only rarely directly addressed by their caregivers [105,106], suggesting that CDC is not essential for language acquisition, at least not as the main source of linguistic experience. A comparative study by Shneidman and colleagues [16] demonstrated that for 1-year-old children growing up in a Yucatec Mayan community, the mean number of utterances a child encountered per hour amounted to approximately 400 utterances, with only 20% of it being directed to the child. The US group of 1 year olds that served as a comparison were exposed to approximately 900 utterances per hour, with more than 70% of these utterances being directed. More recent studies from non-Western, Educated, Industrialized, Rich, and Democratic (WEIRD) [107] cultures confirmed that the amount of directed communication children are exposed to can vary strongly (e.g., the Netherlands: 303 versus Mozambique: 58 utterances of CDC/30 min [108]; Tseltal: 3.63 min of CDC/hour [109]; Tsimane: >1 min/daylight hour [17]; and North American: 11.36 min of CDC/hour [110]), raising questions about the relevance of CDC as the critical source of language acquisition. So far, the factors determining the amount of CDC are unclear. In particular, the role of the child in the society might be crucial, i.e., whether a society adapts situations to the child or expects to the child to adapt to the situation [106,111].

Nonetheless, various studies revealed the presence of CDC features in non-WEIRD cultures (e.g., higher pitch [112]; slower speaking rate [113]; and repetitions, diminutives, and simpler syntax [114]). Overall, the results suggest that both similarities (e.g., in pitch [113]) and differences [115] between WEIRD and non-WEIRD cultures do exist. However, not all CDC features can be found in every culture. In Quiché Mayan, for example, mothers do not seem to produce higher pitch when talking to their children, potentially because they must use this register when speaking to a person of higher status [116].

At this stage, it seems that the only universal characteristic of CDC is the presence of repetitive structural patterns in the input. Clearly, generalizations would be premature until more research reveals patterns linked to the social organization of a linguistic community. However, if one considers CDC as a tool kit, the main features of CDC (Table 1) presumably change gradually as the infant progresses to being a toddler and preschooler [117–120]. During the earliest stage before the 9-month revolution [12] (see Box 1), acoustic and structural features appear to be very prominent, whereas structural features seem to gain greater prominence at later stages (Table 1). Thus, initially, the function of CDC may be to establish and strengthen the social bond with infants, direct attention [121], introduce turn-taking via protoconversations [122], and scaffold the learning of the prosody, phonemes, morphemes, and first words of the local language. After the 9-month revolution, once joint attention, intention reading, symbol recognition, and rational imitation [11] have emerged, CDC may instead be geared more toward the learning of vocabulary and grammar.

A key next step in research would be to determine, for each culture, which features occur at what stage in development and in which combination, and how these tools interact. CDC might turn out to be heterogeneous across cultures. This variation might then be linked to the age at which children achieve adult-level competence in the various components of language.

## The features and functions of immature-directed vocalizations in animals

To identify both the evolutionary roots and adaptive functions of CDC in humans, we must examine similar phenomena in animals. We already noted that preliminary work on great apes suggests our common ancestor featured few, if any, of the elements of CDC as listed in Table 1, at least in the vocal domain. However, it must be stressed that this absence may simply reflect a lack of focused research effort rather than actual absence. But if it is confirmed, this would suggest that surrounding vocalizations provide the primary input for the learned part of the vocal development in great apes and that CDC originated de novo in the human lineage (Fig 1), presumably linked to the emergence of natural pedagogy, which may have preceded, and in fact facilitated, language evolution [53].

We now turn to possible convergent cases. First, we already discussed calls by great ape mothers, but they also occur in other primates [123,124], as well as in many nonprimate species, where mothers call to their infants to retrieve them. Examples include domestic cats (*Felis silvestris catus* [125]), and ungulates such as domestic sheep (*Ovis aries* [126]), cattle (*Bos taurus* [127]), goitred gazelles (*Gazella subgutturosa* [128]), or saiga antelopes (*Saiga tatarica tatarica* [129]). Second, immature-directed calls may serve to aid recognition of the mother’s voice, as in domestic cats [125], Mexican free-tailed bats (*Tadarida brasiliensis mexicana* [130]), fur seals (*Arctocephalus tropicalis* [131]), or domestic sheep [126]. These examples show that even if IDC exists in an animal species, it is unlikely that these cases are functionally equivalent to human CDC.

However, in a third category of species, we find immature-directed calls related to their capacity for vocal accommodation (small alterations of vocalizations as a result of experience [132]) and vocal learning (Box 1). Orcas (*Orcinus orca*) produce family-typical calls at higher rates after the birth of a calf [133]. Likewise, common marmosets (*Callithrix jacchus*), which show evidence of accommodation learning, and thus some level of vocal plasticity [134], modify call rates and repeat various different call types before and after birth of infants [135]. In agile gibbons (*Hylobates agilis*), duetting by mothers with inexperienced young has also been argued to represent IDC, serving to aid the acquisition of the species-specific song [136]. In these cases, the calls may serve to acquire the group’s vocal signature.

Finally, some cases show suggestive parallels to human CDC. In cooperatively breeding marmosets, adults give contingent vocal feedback specifically to infants, which is suggested to impact vocal ontogeny since infants exposed to more of such calls by adults produce and properly use adult-like calls earlier [28,137], possibly owing to increased practice or because vocal feedback reduces stress [13]. This contingent vocal feedback may help infants acquire the underlying rules of dyadic vocal communication (i.e., turn-taking [138], but see [139]). Outside primates, in zebra finches, male tutors use a more stereotypic song when they are near immature birds [140]. In greater sac-winged bats (*Saccopteryx bilineata*), mothers adjust the pitch and timbre when they use immature-directed vocalizations [141].

Despite these parallels, no study has asked exactly which features of the vocalizations (Table 1) are essential and which functions they serve. It is therefore too early to conclude the common incidence of CDC-like functions of immature-directed vocalizations in either primate or nonprimate species [28,40,140–142]. Systematic comparisons are needed to assess the extent of convergence and the determinants, but it remains plausible that IDC serves to facilitate the learning of vocal signatures (in accommodators) or call repertoires (in vocal learners sensu stricto), similar to the language acquisition function of human CDC.

## The function of CDC relative to CSC in humans

Although considerable attention has been paid to CDC and its structuring and function, comparatively less is known about the relative role of surrounding communication that children are exposed to (CSC). Indeed, in some linguistic communities surrounding communication is the primary source of input since adults rarely directly address infants (e.g., Kaluli and Samoan [106]; Yucatec Mayan [16] and Tsimane [17]), at least in their first year of life. Despite these differences in input type, children still become competent native speakers [106,109,143,144]. This inevitably begs the question how important CDC actually is for speech development and suggests that CSC, although currently still underresearched, may have an equally important, perhaps compensatory role in facilitating language acquisition. In small-scale societies, which arguably represent the more typical human condition, children are continuously surrounded by individuals of all ages [145], suggesting that the amount and variation of CSC will be higher than in WEIRD societies. To date, the few studies that to our knowledge have quantitatively assessed this [17,109,146] have not revealed an effect of CSC on vocabulary development [16,101]. However, more work is needed to understand whether CSC supports the learning of other properties of language such as grammatical features.

To obtain a full understanding of how communicative competence develops in both humans and animals, it is critical to account for both sources of input—CDC and CSC—and the interplay between them. Are both CSC and CDC essential for proper language learning, or are they to some extent compensatory? If so, do the large amounts of CDC in WEIRD societies serve to compensate for the much lower quantity of CSC? In animals, immature-surrounding vocalizations might well be the predominant form of input, yet very little research has attempted to quantify their occurrence and assess their influence on the development of communicative competence. Filling this gap should be a high priority for research.

The question arises whether the relative amounts of CDC and CSC seen in humans are comparable to those found in great apes. The one study on chimpanzee infants suggests that immature-surrounding communicative events total approximately 15 gestures, 50 vocalizations, and 3 gesture–call combinations per hour [147]. This is considerably more than what is known so far about the above mentioned low rate of immature-directed vocalizations. In all likelihood, therefore, immature-surrounding vocalizations were the most important source for the learnt part of the vocal system (usage and comprehension learning) in early hominins.

## Conclusions and future directions

In human language learning, the amount and quality of CDC is one of the key facilitators of learning. But how the various features that make up CDC change with age, especially relative to the 9-month revolution, is not clear and should be the target of future studies because they may vary in function from creating attachment, to establishing joint attention, to supporting specific details of language acquisition.

Despite its universality, research across and within cultures has shown enormous variation in a child’s exposure to directed communication. Studies of a few non-WEIRD societies show much lower rates of CDC than found in the typical studies of WEIRD societies. This suggests that the amount of CDC children are exposed to in WEIRD societies might be atypical for the rest of the world and most of human history. Given the fact that all children learn the language of their culture, independent of culture-specific variation in input, the role of CSC for language learning might have been underestimated. The increased amount of CDC in WEIRD societies seems to result mainly in a refinement of skills, involving the size of the vocabulary and the construction inventory involved. This raises the question of how CDC produces this refinement. Its impact may relate to the interactional situations in which it occurs. In these contexts, joint attention is the key component that actually facilitates learning [52,148,149]. Such joint attentional frames allow the reduction of interpretation space of form-meaning associations. Given the extreme cross-linguistic variability of CDC, we must ask the questions of whether and how much CDC is really essential to language learning, whether CSC would do an equivalent job but just more slowly, or whether CDC is essential at particular stages only. Daylong recordings in naturalistic conditions are likely to provide answers to these questions.

To shed light on how CDC evolved, we examined research on our closest relatives, the great apes. So far, very little directed input to infants has been documented. Concerning the features of human CDC (Table 1), few have been found in ape communication, except for repetition of gestures. Repetition is arguably the best predictor of language acquisition in human infants and children [88,89,150]. These findings suggest that short-term repetitive use of communicative acts is potentially an ancestral feature of CDC. We therefore propose that more research is needed on structural repetition to complement the usual emphasis on acoustic features of CDC.

With regard to other animal species, there is more evidence for immature-directed vocalizations in species that engage in vocal learning. This supports the idea that CDC in hominins arose to support the acquisition of highly culturally variable acoustic and structural features of language. However, much more systematic comparisons are needed, which should indicate which of the features characterizing human CDC are also found in these convergent cases. Obviously, more targeted work on great apes is a high priority, if only to see whether repetition is the only CDC-like feature present and why gestures appear to be the exception.

In sum, the current state of research suggests that most features of human CDC have evolved anew in our hominin ancestors. It serves to engage children in social interaction with caretakers and thus to facilitate language acquisition and, in later phases, more explicitly in the acquisition of semantics and grammar. In other words, there is no doubt that CDC is an implicit teaching device. Doubt remains, however, whether it is the only facilitator.

## Abbreviations

CDC: child-directed communication
CSC: child-surrounding communication
IDC: immature-directed communication
